# Treatment of nasopharyngeal carcinoma by tomotherapy: five-year experience

**DOI:** 10.1186/1748-717X-8-107

**Published:** 2013-05-01

**Authors:** Stephen Wan Leung, Tsair-Fwu Lee

**Affiliations:** 1 Department of Radiation Oncology, Kaohsiung Yuan’s General Hospital, Kaohsiung, Taiwan; 2 Department of Radiological Technology, Central Taiwan University of Science and Technology, Taichuug, Taiwan; 3 Department of Electronics Engineering, Medical Physics and Informatics Library, National Kaohsiung University of Applied Sciences, Kaohsiung, Taiwan

## Abstract

**Purpose:**

To analyze of survival curve and toxicity outcomes for patients treated for nasopharyngeal carcinoma (NPC) by intensity-modulated radiotherapy (IMRT) delivered by helical TomoTherapy (HT).

**Materials and methods:**

Since May 2006, 72 patients with primary NPC were treated. In 67 cases PET-CT was used to help delineate the gross tumor volume (GTV); in 4 of these cases distant metastases in bone, mediastinal lymph nodes and unexpected small neck nodes were detected by high SUV uptake. 3, 22, 19, and 27 patients, respectively, had AJCC stage I to IV disease. Patients received a median total dose of 72 Gy to the GTV, 64.8 Gy to the elective PTV, and 54 Gy to the clinically negative neck region.

**Results:**

At a median follow-up of 41 months (range 0.2 to 67 months), no patient has recurred locally. Two patients with stage IIb disease, both of whom received chemotherapy, recurred regionally. Ten patients developed distant metastases. One died from progressive disease with initial proved bony metastasis. Two patients with stage IIb disease, both of whom received chemotherapy, experienced neck node recurrence. 5-year locoregional control rate was 97%; freedom from distant metastases was 84.6% at 5 years. No evidence of disease was detected in 13 early stage (I/IIa/IIb) patients who did not receive chemotherapy. Acute grade 3 toxicity occurred in four patients and grade 4 in two patients. Late toxicities were low, with no grade 3+ xerostomia, grade 2 xerostomia in two patients (3%), and grade 3 hearing loss in two patients (3%).

**Conclusions:**

HT resulted in excellent long-term disease control and survival in heterogeneous NPC patients. Generally mild acute and late toxicity, with low rates of xerostomia, were obtained. Image-guided HT offers the ability to deliver conformal, OAR-sparing dose distributions to a wide variety of NPC patients with good long-term clinical outcomes.

## Introduction

In 2002 the United States Food and Drug Administration (FDA) approved an innovative radiation therapy device called Helical TomoTherapy® (HT; Accuray Incorporated, Sunnyvale, CA). The 3-dimensional imaging provided by megavoltage CT allows for accurate patient setup and precise radiation delivery [1-3]. The helical delivery of intensity-modulated radiotherapy (IMRT) allows excellent conformality and homogeneity of the radiation dose distribution [4-9]. The advantage of its unique approach to radiation delivery is especially clear for complicated dose distributions involving multiple planning target volumes (PTVs) and organs at risk (OAR), such as those often required for adequate and safe treatment of head and neck cancer [5,9].

Considerable research with the HT system has focused on dosimetry of head and neck plans relative to other radiation therapy platforms. In general these studies indicate that HT plans are superior to step-and-shoot (SaS) IMRT plans in terms of target coverage and OAR sparing, or superior to or comparable to arcing IMRT plans, depending on the parameter under consideration. For example, Lee et al. [9] showed that HT plans were more conformal and homogeneous than SaS-IMRT plans, and that the dose to most of the OARs was lower in HT plans (in patients without skull base infiltration HT did not lower the dose to the optic chiasm). Similar outcomes have been obtained in several other studies [6,8,10-12]. Arcing IMRT plans usually fared better than non-arcing IMRT when compared with HT plans; Wiezorek et al. obtained generally superior OAR sparing with arcing IMRT plans in their study of 10 head and neck cancer patients, but concluded the overall plan quality was best for HT.

The first clinical outcomes for HT treatment of head and neck cancer have recently been published. Kodaira et al. [13] used HT to treat 20 pts with NPC, some node-positive and with metastases, most of whom underwent chemotherapy. The progression-free and overall survival rates at 10 months were 79.7% and 95%, respectively. Parotid function assessed by quantitative salivary scintigraphy showed a drop in maximal excretion ratio at 3 months which returned to near baseline by one year; at 3 months 61.1% of patients had Grade 2 and 38.9% had Grade 1 xerostomia, but by 9 months, Grade 2 xerostomia was observed in 26.7% of patients, Grade 1 in 66.7%, and Grade 0 in the rest. Most recently Chen et al. [14] studied patients treated for nasopharyngeal cancer (NPC) using HT or IMRT delivered via segmental multileaf collimator. They replicated the dosimetric superiority of HT, showing significant reductions in dose to parotids, temporal lobes, and ipsilateral ear structures. Overall survival and disease control for the two groups was nearly identical. Although RTOG-graded scoring of late xerostomia was not significantly different (13% for IMRT vs 7% for HT), 38% of IMRT patients (7 of 16 patients) reported “too little” or “no” saliva using the University of Washington quality of life survey (UW-QOL), while 7% (1 of 14 patients) of HT patients reported too little saliva. The authors concluded that the dosimetric differences between HT and SaS-IMRT did, indeed, result in improved clinical outcomes. Despite these instances, published clinical data on HT for NPC is sparse. Here we present long-term (5 year) clinical outcomes of HT for 72 patients with nasopharyngeal cancer.

## Materials and methods

### Patients

Since May 2006, 72 patients (59 men and 13 women) with primary NPC were treated with curative RT by HT in the Department of Radiation Oncology, Kaohsiung Yuan’s General Hospital. Patient characteristics are indicated in Table 1. The median age was 46.5 years (range 23 to 80 years). The distribution of clinical stages according to the American Joint of Cancer Committee (AJCC) staging system published in 1997 was 3 patients (4%) at Stage I, 23 (32%) at Stage II, 19 (26%) at Stage III, and 27 patients (37.5%) at Stage IV. Forty-five patients (62.5%) received concomitant chemotherapy, and 27 patients (37.5%) did not. This study was approved by the Medical Ethics Committee of the Kaohsiung Yuan’s General Hospital (YUAN-IRB20120328B). Written informed consent was obtained from the patient for publication of this report and any accompanying images.

**Table 1:** Patient characteristics (n = 72)

**Variables**	**Number (%)**
Age, median years (range)	46.5 (23–80)
Gender	
Male	59 (82%)
Female	13 (18%)
Smoker	
Yes	30 (41.5%)
No	40 (55.5%)
Unknown	2 (3%)
AJCC stage	
I	3 (4%)
II a/b	7/16 (total 32%)
III	19 (26%)
IV a/b/c	6/14/7 (total 37.5%)
T stage	
T1	30 (41.5%)
T2	11 (15%)
T2a	2 (3%)
T2b	6 (8%)
T3	15 (21%)
T4	8 (11%)
N stage	
N0	13 (18%)
N1	24 (33%)
N2	20 (28%)
N3	15 (21%)
Combination with chemotherapy	
Yes	45 (62.5%)
No	27 (37.5%)
Brachytherapy	
Yes	15 (21%)
	Stage I: 1 patients
	Stage IIa 3 patients
	Stage IIb 7 patients
	Stage III 4 patients
No	57 (79%)
PET/CT fusion	
Yes	67 (93%)
No	5 (7%)

AJCC, American Joint of Cancer Committee in 2007.

### Treatment planning

All patients were immobilized in a tailor-made thermoplastic cast from head to shoulders, and 3 mm-thickness CT (12 patients) and/or PET/CT (67 patients; Siemens Biograph LSO PET/CT) scan slices of the head and neck were obtained to be used for localization of targets and organs at risk (OARs). CT or PET/CT image sets were then transferred to and fused in the Pinnacle^3^ planning system (Phillips Healthcare, The Netherlands) for contouring. Contours were transferred to HT for IMRT inverse planning. In four of the patients simulated with PET/CT, distant metastases in bone, mediastinal lymph nodes and unexpected small neck nodes were detected by high SUV uptake.

The gross tumour volume (GTV), including the macroscopic primary cancer and nodes greater than 1 cm in diameter or nodes with necrotic centres, was used for all plans. RT was delivered using a sequential IMRT technique and three cone-down target volumes were defined for each patient. According to the RTOG 0225 and previous studies [15-18], for a typical case by using the prescribed dose equals to 72 Gy, the clinical target volume (CTV) for the delivery of absorbed dose of 72 Gy (CTV_72_ = GTV + 5 mm margin) is defined using an isotropic margin of 5 mm around the GTV. The clinical target volume for 64.8 Gy delivery (CTV_64.8_) equals to the CTV_72_ + 5 mm margin plus areas at risk for microscopic involvement, including the entire nasopharynx, retropharyngeal nodal regions, skull base, clivus, pterygoid fossae, parapharyngeal space, sphenoid sinus (in T3-T4 disease, the entire sphenoid sinus), the posterior third of the nasal cavity/maxillary sinuses that includes the pterygopalatine fossae, and levels I through V nodal regions. The cavernous sinus should be included in high-risk patients (T3, T4, bulky disease involving the roof of the nasopharynx). The clinical target volume for 54 Gy (CTV_54_) includes the clinically negative low neck regions. Safety margins between the CTV and planning target volume (PTV) of 5 mm were used for CTV_72_ and CTV_64.8_ to account for patient setup error and motion uncertainties, but in areas in which the GTV or the CTV was adjacent to critical normal structures (i.e., the brainstem) the margin was reduced to 1 mm; no safety margin was used for the generation of planning target volume-54Gy (PTV_54_). In our study cohort, the median (and modal) prescribed dose was 72 Gy to the PTV (39.6 Gy to 75.6 Gy) (PTV_72_), 64.8 Gy to the elective PTV(PTV_64.8_), and 54 Gy to the clinically negative neck region (PTV_54_) with a daily fraction size of 1.8 Gy in terms of three cone-down treatment schemes. Typically for the prescribed dose equal to 72 Gy, the prescription dose was set to (a) 28 fractions containing all three PTVs, (b) eight fractions containing PTV_72_ and PTV_64.8_, and (c) four fractions treated with PTV_72_ alone. OARs included seven serial-type organs (serial OARs; brainstem, spinal cord, lenses, eyes, optic nerves, chiasm, and mandible) and three parallel-type organs (parallel OARs; parotids, submandibular glands, and oral cavity). Maximum doses to OARs were optimized on an individual basis without compromising the PTV coverage, with at least 95% of the PTV receiving the minimum prescribed dose. Fifteen patients (21%) also received intracavitary brachytherapy delivered to the GTV, 3–6 Gy in 1–2 fractions delivered twice per week, the dose prescribed to 2 cm off the source axis.

### Treatment delivery

Treatment was delivered in five fractions per week. Daily patient setup began with laser-based positioning followed by MVCT scanning using the coarse setting. Offsets were used to correct patient positioning. IMRT was delivered with a 2.5 cm field width, a pitch of 0.3, and a modulation factor of 2.5. These settings have been shown in prior work to produce plans that are superior to 7-field step-and-shoot IMRT plans [9]. Repeat CT simulation occurred 2 to 3 times during treatment to allow cone-down boosting and plan adaptation to meet dosimetric goals for PTVs and maintain dose constraints to OARs.

Concurrent chemotherapy was administered in 45 (62.5%) patients. For most patients this consisted of cisplatin and fluorouracil; some patients were treated with combinations that included oxaliplatin, tegafur-uracil (Ufur), hydroxyurea, carboplatin, or erbitux.

### Patients follow-up

Patients were evaluated for disease control, survival, and toxicity according to RTOC toxicity criteria at 2-month intervals for the first 2 years, at 3- to 6-month intervals between the third and fifth years, and at 1-year intervals thereafter. Follow-up examination of the primary tumor was assessed by fiberoptic endoscopy at every visit. CT scan, bone scans, chest radiography, and liver sonography were performed every 6 months in the first 2 years and annually thereafter.

## Results

### Dosimetry

Highly conformal, homogeneous treatment plans were generated for HT [9]. Table 2 shows that dose constraints for organs at risk were generally maintained. In particular, both ipsilateral and contralateral parotid glands were held below the 26 Gy dose constraints. Doses to OARS were always less than doses recommended under RTOG protocol 0615.

**Table 2:** The mean or maximum doses to organs at risk (OARs)

**OARs**	**Mean value ± SD (Gy)**	**Range (Gy)**
Serial	Maximum dose	
Brain stem	50.53 ± 1.78	46.40–52.75
Spinal cord	39.95 ± 1.95	35.86–42.90
Ipsi-lateral lens	3.78 ± 0.78	2.63-4.70
Contra-lateral lens	3.58 ± 0.70	2.60-4.60
Ipsi-lateral eye	11.19 ± 3.10	6.76-17.69
Contra-lateral eye	10.38 ± 2.51	6.53-15.86
Mandible	63.64 ± 1.72	60.17-65.44
Chiasm	38.20 ± 4.67	15.20-46.50
Parallel	Mean dose	
Ipsi-lateral parotid gland	22.07 ± 1.66	19.18-24.99
Contra-lateral parotid gland	20.46 ± 2.27	15.48-24.56
Submandibular glands	31.92 ± 2.65	27.85–37.09
Oral cavity	32.49 ±6.09	22.19–41.06

### Disease control

**Figure 1:**
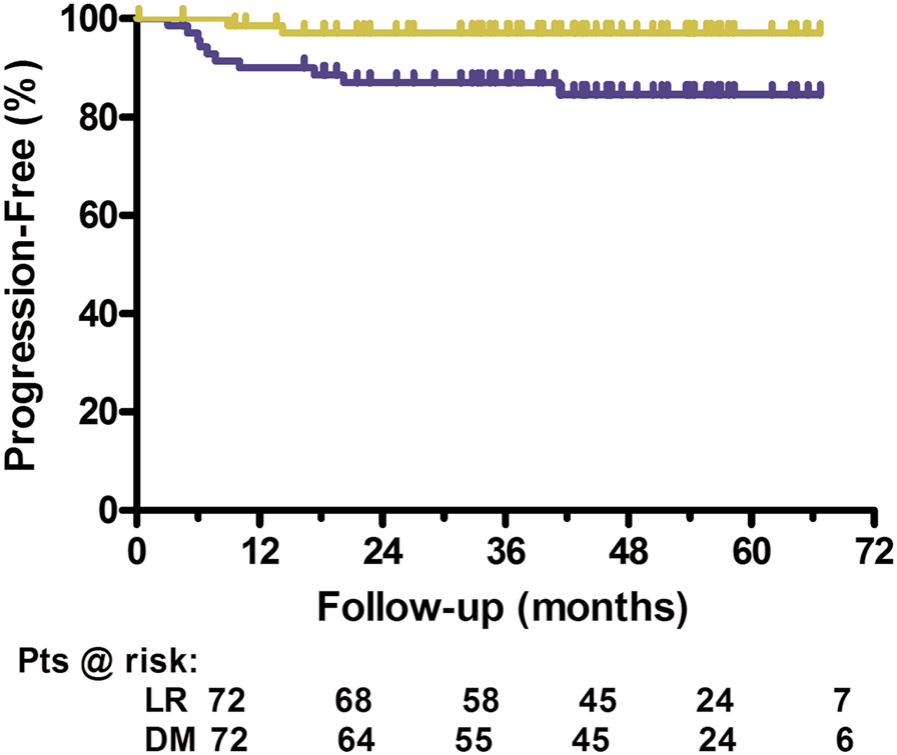
**Locoregional progression-free survival (yellow) and freedom from distant metastases (blue).** The 5-year locoregional control estimate was 97% (95% CI = 88.7% – 99.2%). KM freedom from distant metastases was 84.6% at 5 years (95% CI = 72.9% – 91.5%). Below the figure is shown the patients at risk at different time points; LR indicates locoregional DM indicates distant metastases.

At a median follow-up of 41 months, no patient has recurred locally. Two patients with stage IIb disease, both of whom received chemotherapy, experienced neck node recurrence. Kaplan-Meier (KM) analysis resulted in a 5-year estimate of locoregional control of 97% (see Figure 1). One of these patients was treated surgically and is currently without evidence of disease; the other was lost to follow-up. Ten patients had been proved for distant metastasis; KM freedom from distant metastases was 84.6% at 5 years (Figure 1). Metastases occurred to bone, lung, and liver, and 5 patients presented with multiple metastatic sites. One patient died for disease out of control with initial proved bony metastasis. No evidence of disease was detected in 13 early stage (I/IIa/IIb) patients who did not receive chemotherapy. Seven patients have died of cancer; overall survival at 40 months mean follow-up was 90%.

### Toxicity

Most patients experienced no serious acute toxicity (Table 3). RTOG Grade 2 acute mucositis was observed in 6 patients (8%), Grade 3 in 3 patients (4%), and Grade 4 in 1 patient (1%). Grade 2 dermatitis occurred in 8 patients (11%), Grade 3 in no patients, and Grade 4 in 1 patient (1%). All patients with Grade 3 and 4 acute toxicities were also treated concurrently with chemotherapy. Late toxicity was generally very mild, with Grade 2 xerostomia in 2 patients (3%) and no Grade 3+ xerostomia. Grade 2 dysphagia occurred in 1 patient, with no Grade 3+ dysphagia. Grade 3 hearing loss was observed in 2 patients (3%) who also received concurrent chemotherapy.

**Table 3:** Acute and late toxicity (RTOG grading criteria)

	**Grade**				
**Acute toxicity (N/%)**	**0**	**1**	**2**	**3**	**4**
Mucositis	42 (58)	20 (28)	6 (8)	3 (4)*	1 (1)*
Dermatitis	13 (18)	50 (69)	8 (11)	0 (0)	1 (1)**
Late Toxicity (N/%)	0	1	2	3	4
Xerostomia	21 (29)	49 (68)	2 (3)	0 (0)	0 (0)
Dysphagia	65 (90)	6 (8)	1 (1)	0 (0)	0 (0)
Hearing loss	35 (49)	34 (47)	1 (1)	2 (3)	0 (0)
Other	72 (100)	0 (0)	0 (0)	0 (0)	0 (0)

*Patients received cisplatin, fluorouracil, Ufur.

**Patient received oxaliplatin.

## Discussion

The dosimetric advantages of HT for head and neck cancer have been reported in numerous publications, but few studies report on clinical outcomes of HT. The current study is among the largest, with the longest follow-up, reported in the literature to date. Its results demonstrate outstanding long-term disease control with acceptable levels of toxicity for NPC treated by NPC.

By allowing safe dose escalation to high-risk regions and simultaneous broad-field treatment of low-risk regions, IMRT has, arguably, enhanced NPC treatment efficacy and safety [17]. Lee et al. reported results from a multi-institutional trial of 68 patients with NPC treated with IMRT with or without chemotherapy [19]. Most patients received 70 Gy to the GTV and 59.4 Gy to subclinical regions. At a median follow-up of 2.6 years the 2-year estimate of local control was 92.6%, progression-free survival was 72.7%, and overall survival 80.2%. Grade 4 acute toxicities were observed in 11.8% and Grade 3 in 61.8% of patients. Grade 2 xerostomia at 1 year was present in 13.5%. Wolden et al. [20] treated 74 patients to 70 Gy with GTV boosting; most patients received concurrent and adjuvant platinum-based chemotherapy. At a median follow-up of 35 months the 3-year actuarial local control estimate was 91%; progression-free survival and overall survival was 67% and 83%, respectively. Long-term Grade 2 xerostomia occurred in 32% of patients (no Grade 3). Similar rates of long-term local disease control and overall survival, with Grade 2–3 xerostomia related to parotid dose [21], were reported by others using IMRT [21,22].

More recently reports of HT for locally advanced NPC have begun to appear. Kodaira et al. [13] treated 20 patients to 70 Gy to the tumor PTV and 54 Gy to the nodal PTV in 35 fractions. At a median follow-up of 10.3 months, 2 patients developed recurrent disease, in the liver in one patient and in a lymph node included in the tumor PTV for another. Progression-free and overall survival was 79.7% and 95%, respectively. Shueng et al. [23] followed 28 patients to a median of 33 months after neoadjuvant chemotherapy followed by concurrent chemoradiation including HT to 70 Gy to the tumor and positive nodes. The 3-year local, regional, locoregional control and distant metastasis rate were 92.4%, 95.7%, 88.4%, and 78.0%, respectively, and overall survival was 83.5%. Toxicities generally occurred at a low rate, and late Grade 2 xerostomia was observed in 14% of patients (no Grade 3+). Ren et al. [24] treated 73 patients, 24 with radiation only, with 70–74 Gy in 33 fractions to tumor PTV (primary tumor and positive lymph nodes), with 60–62.7 Gy a high-risk and 52–56 Gy to a low-risk PTV. At a median follow-up of 14.8 months the 1-year relapse-free survival, distant metastasis-free survival and overall survival were 95.6%, 97.2% and 94.8%, respectively. By one year post-radiotherapy no patient reported Grade 2+ xerostomia. In this context the current results compare favorably; at a median of 41 months follow-up, with several patients followed beyond 4 and 5 years, no patient recurred locally. Two regional recurrences and 10 distant metastases were obtained, and to date seven deaths due to cancer has occurred. Mean parotid doses were very low—22.07 Gy and 20.46 Gy to the ipsilateral and contralateral glands, respectively—and late Grade 2+ xerostomia occurred in only 3% of patients. We conclude that the ability of HT to effectively target multiple regions of differing disease burden within the head and neck field while maximally sparing OARs, combined with daily MVCT-guided patient setup accuracy and a rational regimen of systemic treatment, results in robust disease control with limited toxicity.

Although local control and survival have been enhanced by the introduction of IMRT into NPC treatment, the pattern of recurrence continues to show a relatively high rate of distant metastases [17,20,21,23], suggesting the continuing need for more effective systemic therapies. In addition, it cannot be determined whether the generally superior dose-sculpting capability of HT relative to more conventional IMRT leads to improved disease control and toxicity outcomes. A recent paper by Chen et al. [14] reported on a comparison of 16 patients treated with IMRT delivered by sequential multileaf collimator and 14 with HT. Survival and disease control were identical between groups; 2-year local control was 87% in both groups, and overall survival did not differ significantly. The rate of xerostomia at any point in the late setting did not differ significantly between groups; at last follow-up, however, 38% of IMRT patients reported “too little” or “no” saliva, versus 7% of HT patients. Thus, recovery from xerostomia occurred more readily in the HT group. In a study by our group of quality-of-life between IMRT and HT patients, substantial improvements in saliva and oropharyngeal QOL variables were obtained with HT (in preparation). Thus, although for many patients superior HT treatment plans may have little effect on outcomes, for a significant subset the ability to easily reduce dose to parotids and other structures can be important for long-term QOL.

Potential limitation for this study may exist. Xerostomia related symptoms were usually cited as the most prevalent complications in NPC survivors post radiotherapy and patient-reported xerostomia has been found to significantly correlate with mean dose to the parotid glands and the minor salivary glands. Assessment of treatment-related toxicity by the well-validated health-related quality of life questionnaire rather than RTOC toxicity criteria would provide more informative data for the comparison among different studies.

The outcomes of the present study require additional long-term follow-up to assess the robustness of the promising disease control and low toxicity observed to date. In addition, the single-institution nature of the current study suggests a need to expand the HT methodology employed here to different patient populations in different institutions. In the meantime we are encouraged by the relatively low toxicity and excellent disease control with HT and, in selected patients, chemotherapy, to carry on with this approach to treating patients with locally advanced nasopharyngeal carcinoma.

## Conclusions

Modern, advanced technology has brought the radiation oncology of head and neck cancer to a new era. We can generate better conformity and homogeneity than ever. In our clinical practice, acute and late complications after HT for NPC are definitely fewer and milder compared with our conventional IMRT technique. Helical TomoTherapy appears to have the advantages compared to IMRT for NPC treatment plans in terms of dosimetric distribution, and quality of life, local control and survival for our patients. Nevertheless, HT appears to have dosimetric advantages compared to IMRT which might translate into a significant benefit for the patients in clinical practice. To our knowledge, charged particles, such as proton beam or carbon ion beam, may have the best dosimetry for NPC plans. Comparison of the treatment outcomes of TomoTherapy to that of charged particles would be an interesting issue and warrants further investigation.

## Competing interests

The authors declare that they have no competing interests.

## Authors’ contributions

SWL: original idea, study design, data collection and writing of manuscript. TFL: study design, statistical analysis, technical supports and correspondence. Both authors read and approved the final manuscript.
